# Supplementary material for: In-situ liquid cell transmission electron microscopy investigation on oriented attachment of gold nanoparticles
Source: Nat Commun. 2018 Jan 29;9:421. doi: 10.1038/s41467-018-02925-6 (PMC5788991; doi:10.1038/s41467-018-02925-6)
Supplement: Supplementary file 1 — Supplementary Information [file 41467_2018_2925_MOESM1_ESM.pdf]

In-situ Liquid Cell Transmission Electron Microscopy Investigation on Oriented Attachment of Gold Nanoparticles

Chao Zhu[1], Suxia Liang[2], Erhong Song[3], Yuanjun Zhou[4], Wen Wang[1], Feng Shan[5], Yantao Shi[2], Ce Hao[2], Kuibo Yin[1], Tong Zhang[5], Jianjun Liu[3], Haimei Zheng[6⋆], Litao Sun[1,7,8⋆]

[1]*SEU-FEI Nano-Pico Center, Key Laboratory of MEMS of Ministry of Education, Collaborative Innovation Center for Micro/Nano Fabrication, Device and System, Southeast University, Nanjing 210096, P. R. China.*

[2]*State Key Laboratory of Fine Chemicals, School of Chemistry, Dalian University of Technology, Dalian 116024, P. R. China.*

[3]*The State Key Laboratory of High Performance Ceramics and Superfine microstructure, Shanghai Institute of Ceramics, Chinese Academy of Sciences, Shanghai 200050, P. R. China.*

[4]*Department of Physics and Astronomy, Rutgers, The State University of New Jersey, Piscataway, NJ 08854, USA.*

[5]*Joint International Research Laboratory of Information Display and Visualization, School of Electronic Science and Engineering, Southeast University, Nanjing 210096, P. R.*

[6]*Materials Sciences Division, Lawrence Berkeley National Laboratory, Berkeley, CA 94720, USA.*

[7]*Center for Advanced Carbon Materials, Southeast University and Jiangnan Graphene Research Institute, Changzhou 213100, P. R. China.*

[8]*Center for Advanced Materials and Manufacture, Joint Research Institute of Southeast University and Monash University, Suzhou 215123, P. R. China.*

⋆Correspondence author. E-mail: slt@seu.edu.cn or hmzheng@lbl.gov

Supplementary Figures

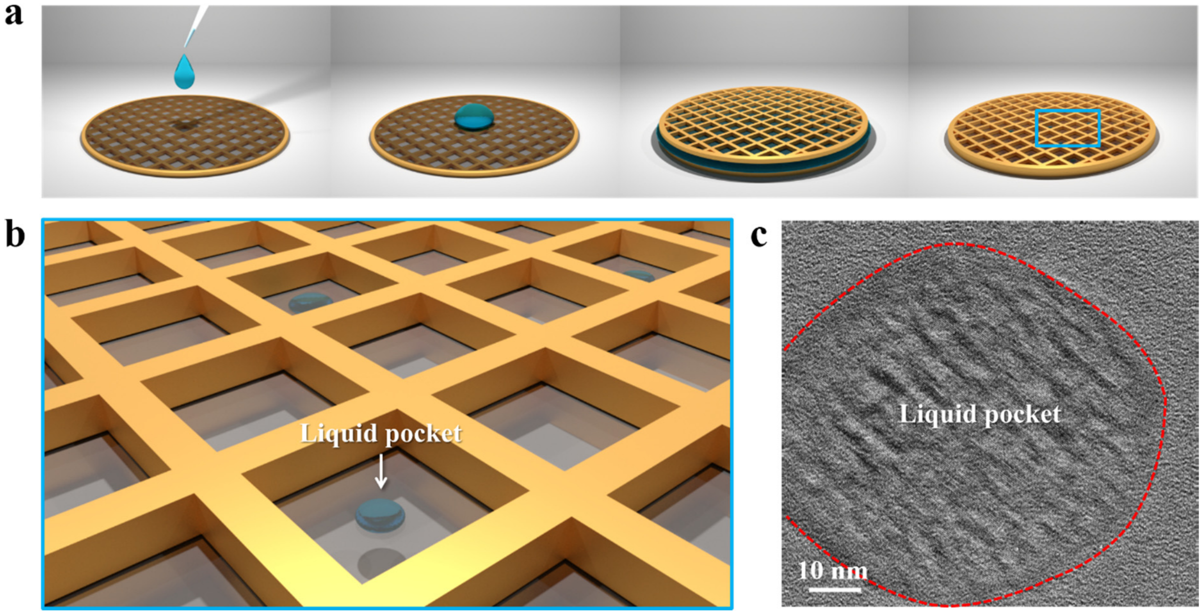

Supplementary Figure 1. Preparation of a carbon film based liquid cell. **a** Schematic illumination for the fabrication of liquid cell: A droplet of as-prepared solution was sandwiched by two TEM copper grids with formvar stabilized carbon support films face-to-face. The sample was then left under atmosphere until the extra liquid volatized. **b** An enlarged scheme shows the liquid encapsulated by carbon films. **c** TEM image of a sealed liquid pocked with the size of 80 nm.

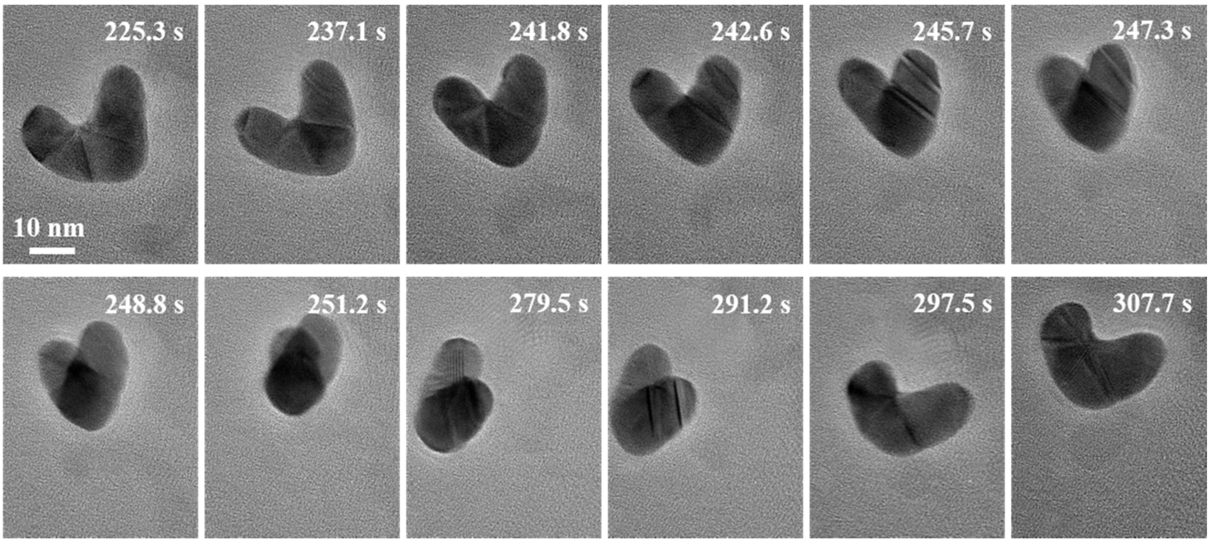

Supplementary Figure 2. Free rolling of "L" shape nanostructure. To ensure the occurrence of OA is in solution rather than in vacuum, we have monitored the movement behaviors of different large particles. This figure is the TEM sequences to show the motion of a "L" shape nanostructure in the liquid pocket. It can be seen that the particle undergo free translation and rotation in solution. It does not lay on the carbon films, but keeps rolling freely (not only along the viewing axis but also off axis) because it is suspended in solution.

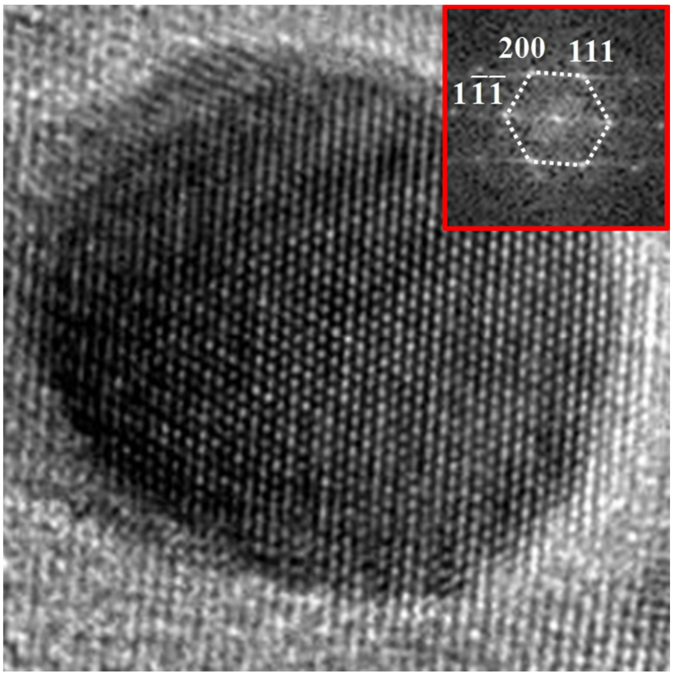

Supplementary Figure 3. High resolution TEM image frame of a large gold nanoparticle. A still snapshot from Supplementary Movie 1 of large gold nanoparticle exhibits the atomic resolution of our carbon film based liquid cell. The clear lattice fringes and inserted FFT indicates the viewing direction is $[0\bar{1}1]$ zone axis.

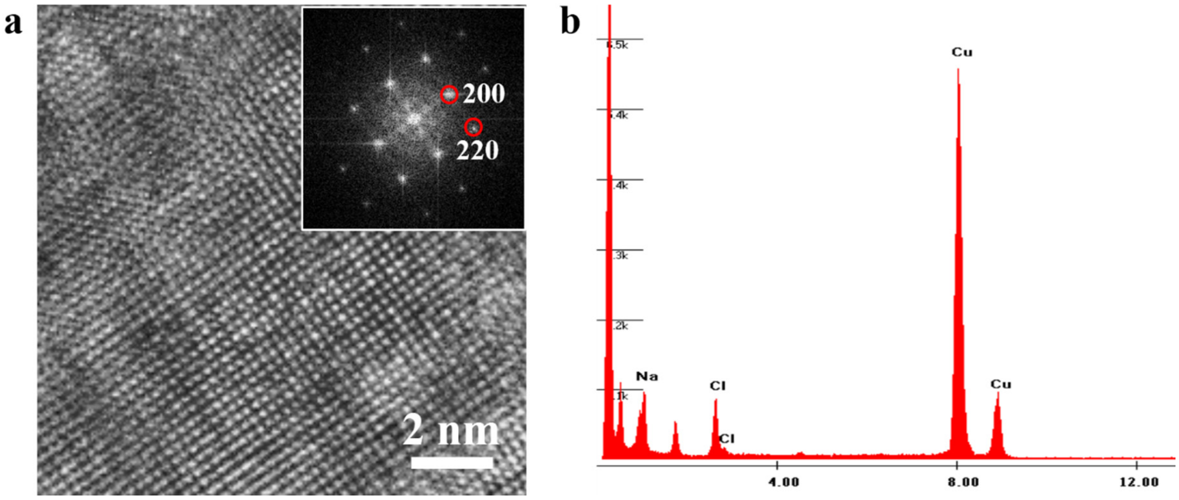

Supplementary Figure 4. Characterization of NaCl crystals. **a** A high resolution TEM image of well crystallized NaCl with nearly no defects. The inserted FFT of this image shows the reciprocal lattice points (200) and (220), in accordance with 0.28 and 0.20 nm d-spacing of NaCl. This image is recorded at location of a liquid pocket, which indicates that the observed solid-state NaCl is precipitation from supersaturated solution and adsorbed onto surfaces of carbon films. **b** EDX result confirms that the components of this material are only Na and Cl elements.

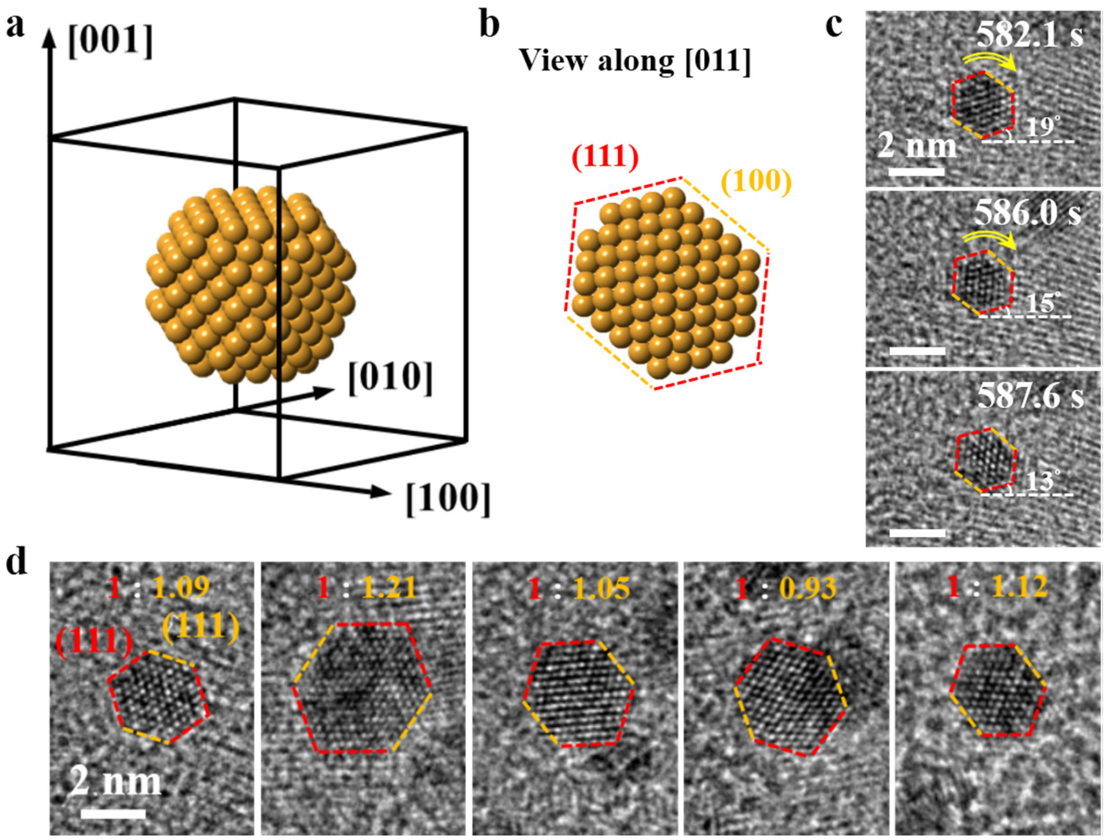

Supplementary Figure 5. Nanoparticle morphology for tracking of their motions and orientations, as well as measurement of their surface area. **a** The equal proportional atomic model of a truncated gold nanoparticle. Theoretical calculations have predicted that gold nanoparticles in the 1 − 3 nm size range, prefer to keep a truncated octahedral or cubeoctahedral appearance rather than other symmetries such as Ino-decahedral and icosabedral[20-22]. **b** 2D projection of the model viewed along [011] zone axis. **c** TEM sequences show the motion trajectories of a particle. The hexagonal morphology of this particle along [011] zone axis confirms its truncated octahedral 3D constructions. Thus, rotation of the particle can be accurately monitored by a combination of its morphology and crystal orientation. As an example here, the particle clockwise rotate about 6 degree during 5.5 seconds (Arrows indicate the rotation direction and white dashed lines are horizontal references). Moreover, we find that the surfaces of particles smaller than 3 nm are all composed of {111} and {100} surfaces, but no any obvious {110} surfaces. It is reasonable because in such a small size, surface energy may contribute more than volume energy, so the unstable {110} surface with highest energy is not a favorable surface. **d** Snapshots captured from videos show that most of the small particles have a highly symmetrical truncated octahedron morphology, which is composed of 8 equivalent {111} surfaces and 6 equivalent {100} surfaces as illustrated in (a). Then we estimated their surface area for each particle and the ratio between {111} and {100} is shown at the top of images, ranging from about 1 : 1.2 to 1 : 0.9.

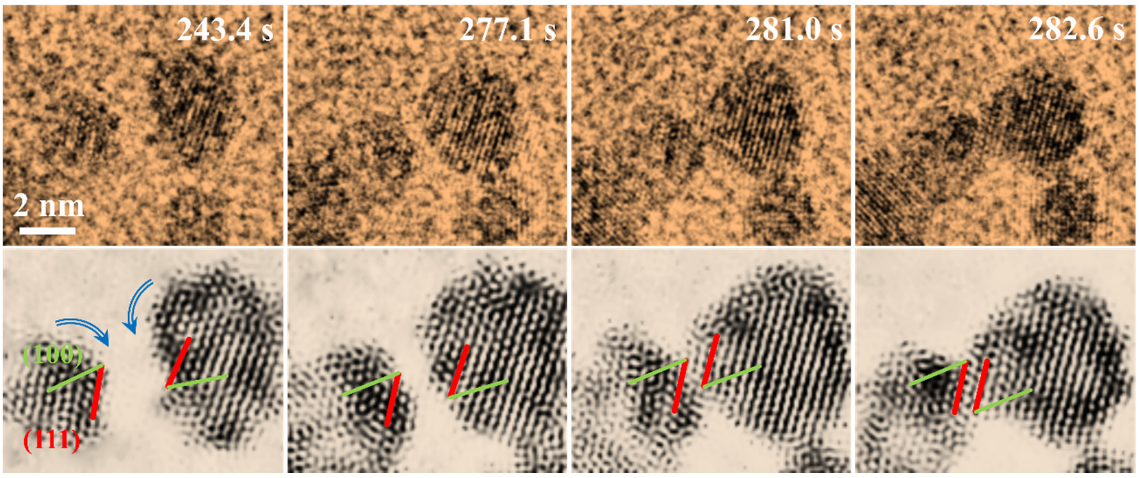

Supplementary Figure 6. A single crystal structure formed through oriented attachment. Image sequences show the OA process of gold nanoparticles at {111} surfaces. The contact at their aligned {111} surfaces with parallel {100} facets lead to a final single crystal structure.

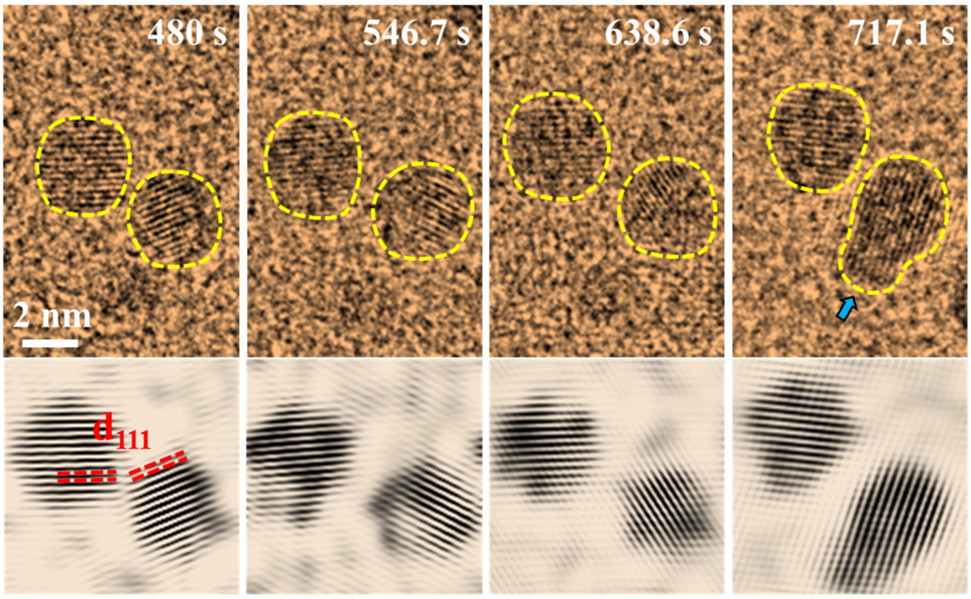

Supplementary Figure 7. A particle pair with on occurrence of OA. The particle pair is close enough for more than 230 seconds, during which the right particle keeps rotating but still fails to get a pre-alignment of their {111} facets. Finally, it coalesces with another incoming particle (noted by the blue arrow) instead of the left one to form a perfect single crystal structure. The lower rows are corresponding filtered images.

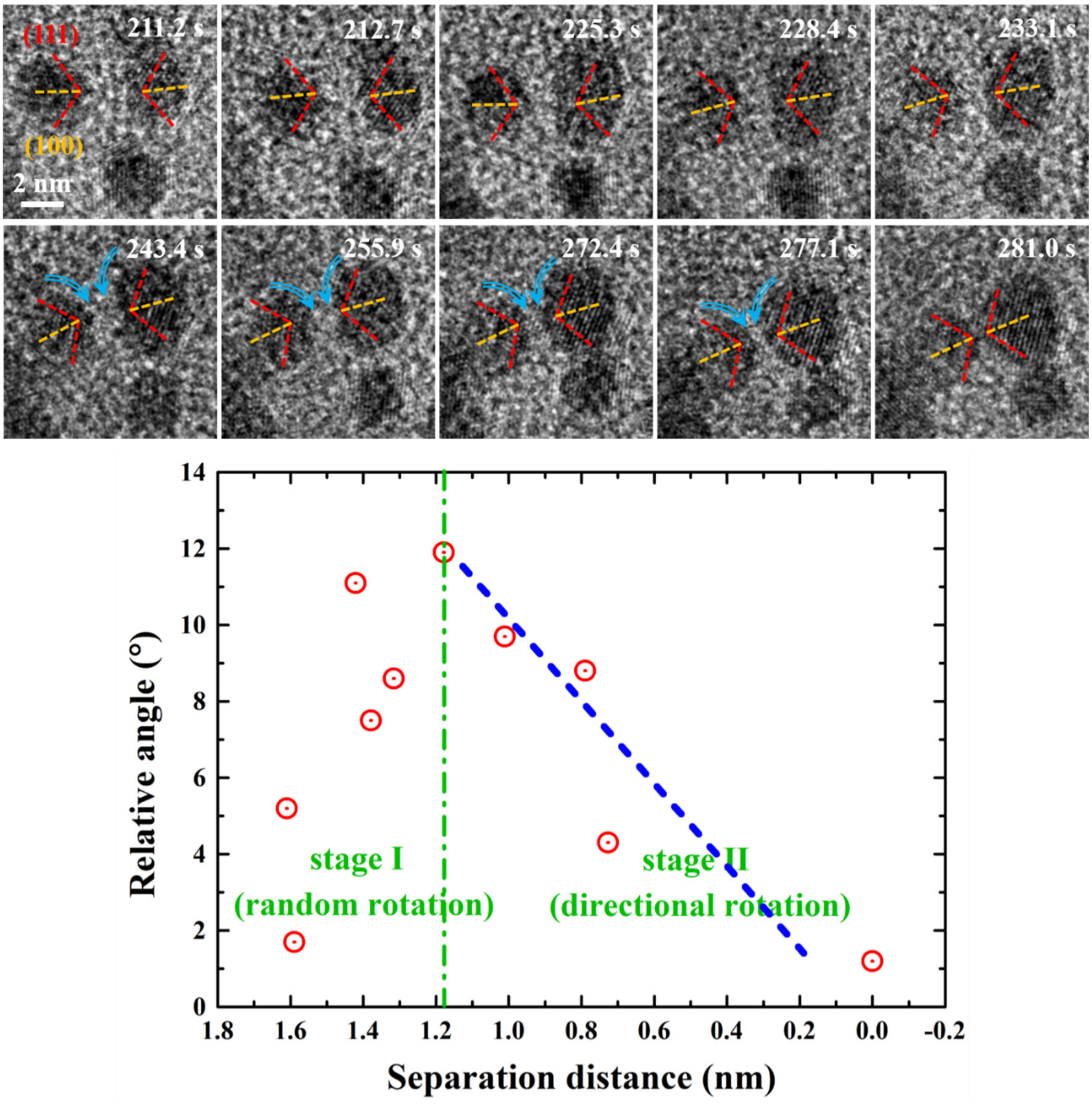

Supplementary Figure 8. OA process and the changes of their relative angle between {111} facets of a particle pair. In this case, the angle changes randomly when D > 1.2 nm. Then as the particle pair gets closer (D > 1.2 nm), the directional rotation begins to make the angle gradually decrease to approximate 0º.

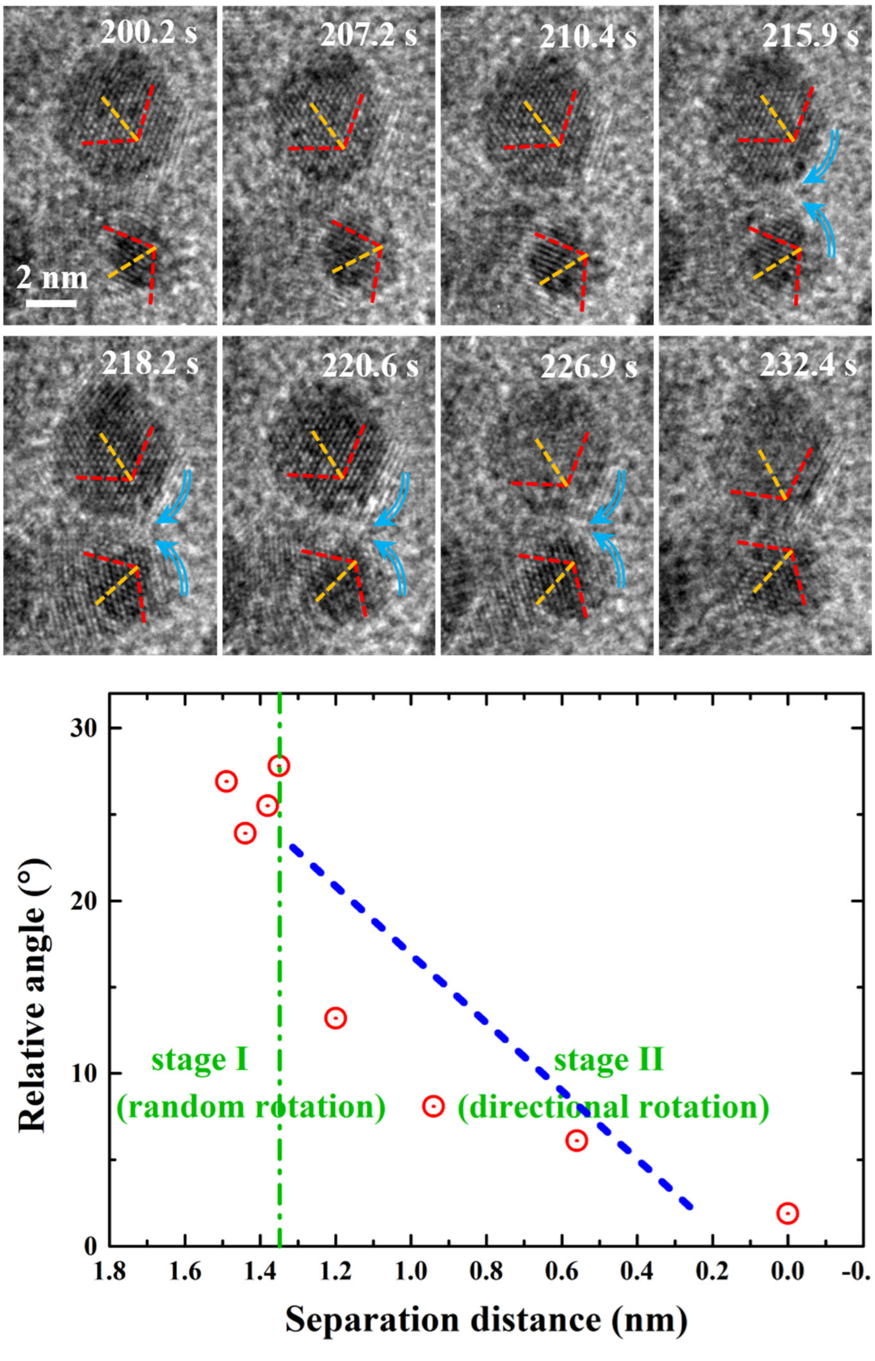

Supplementary Figure 9. OA process and the changes of their relative angle between {111} facets of a particle pair. In this case, the angle does not change much as $D > 1.3$ nm. But when the particle pair approaches to a smaller distance ($D < 1.3$ nm), it quickly decreases to nearly 0º.

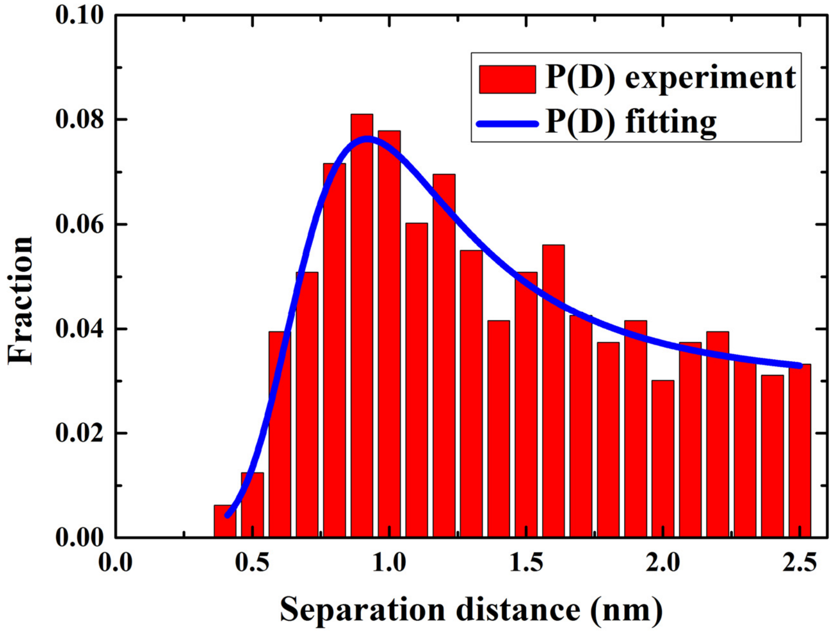

Supplementary Figure 10. Distribution of separation distance. Statistical distribution of the separation distance for the observed OA events by 21 particle pairs and the fitting result using Boltzmann distribution as given in Supplementary Equation (5).

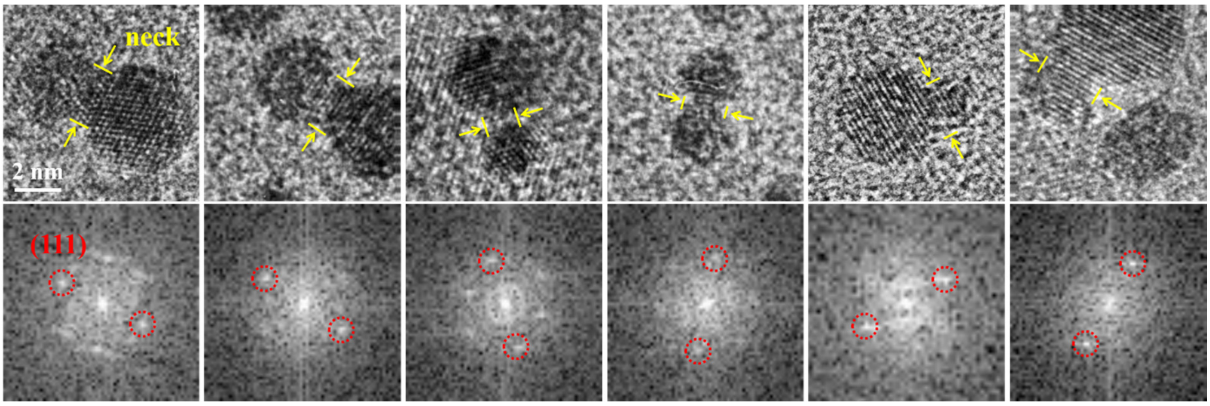

Supplementary Figure 11. Oriented attachment at {111} surfaces. TEM snapshots from videos show the contact moment of different particle pairs, illustrating that all OA events occur at their {111} surfaces. The lower sequences are corresponding FFT images of the neck location of each particle pair. The d-spacing is confirmed to be 0.24 nm for Au {111} facets.

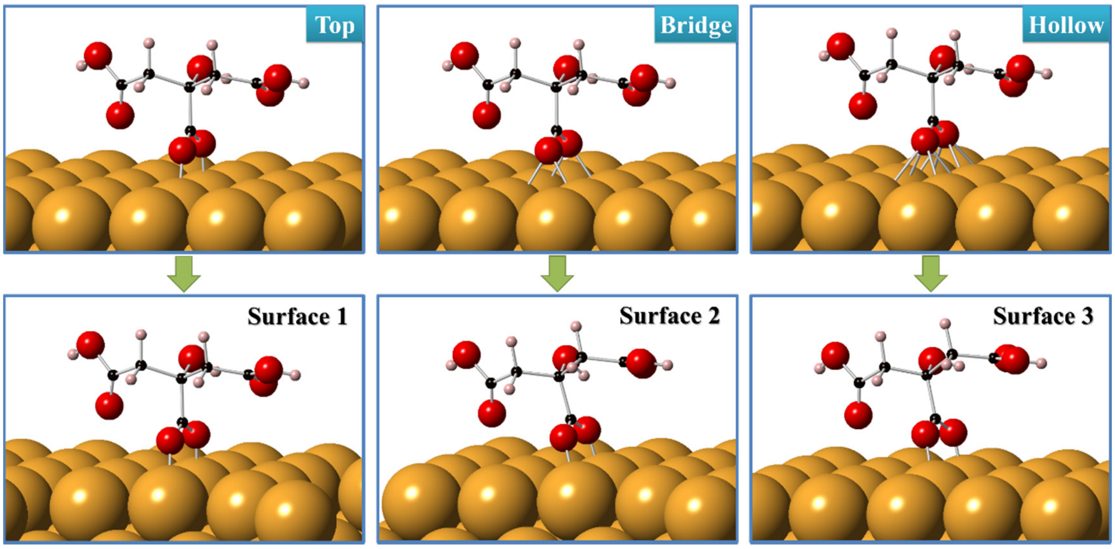

Supplementary Figure 12. Ligand configurations on {100} surfaces. Adsorbed citrate configurations on gold {100} surfaces before (upper row) and after (lower row) relaxation.

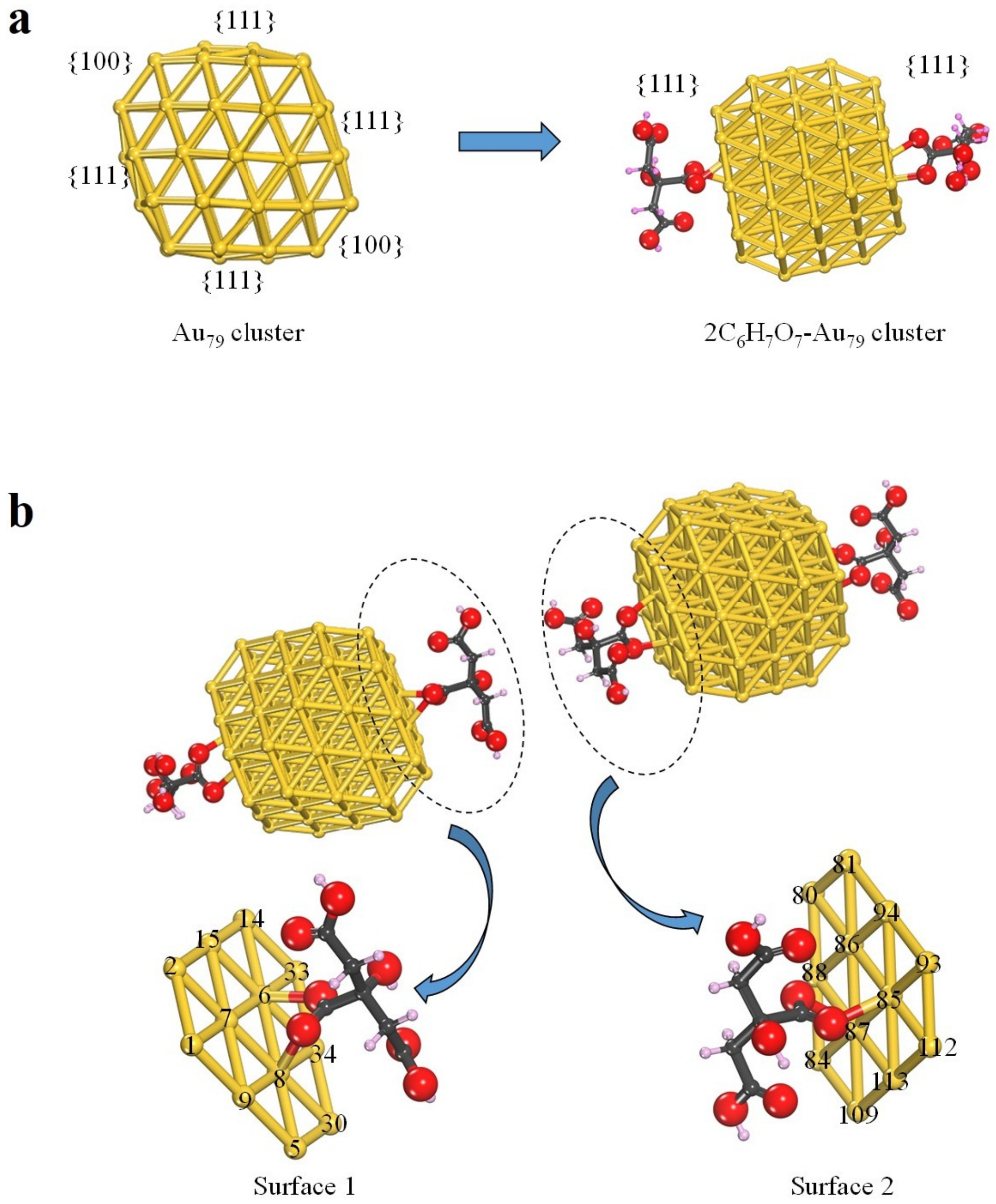

Supplementary Figure 13. Configuration of citrate ligands adsorbed on {111} surfaces of Au_{79} particles during Bader charge calculation. **a** Simulation of relaxation of multifaceted Au_{79} particles with a size of 1.3 nm, and two citrate ligands symmetrically adsorb on {111} surfaces. **b** Simulation of relaxation of two attaching Au_{79} particles with ligands. Ligands adsorb on {111} faces of surfaces 1 and 2, respectively. Atoms are noted with colors (yellow: gold; red: oxygen; gray: carbon; white: hydrogen).

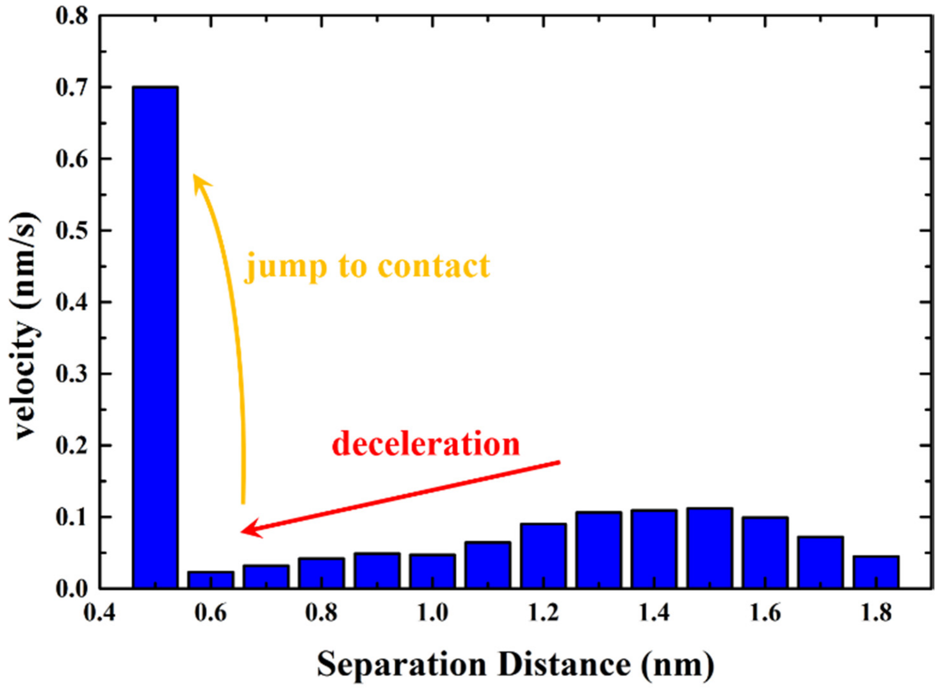

Supplementary Figure 14. Relative drift velocity of a particle pair when they approach each other.

Supplementary Tables

Bader charge of Au atoms on surface 1 {111}												
D (nm)	1	2	5	6	7	8	9	14	15	30	33	34
1.5	11.056	11.050	11.053	10.829	10.875	10.836	11.008	11.038	10.987	11.067	11.030	10.967
1.3	11.056	11.051	11.053	10.830	10.875	10.837	11.007	11.039	10.987	11.068	11.031	10.968
0.9	11.056	11.051	11.051	10.829	10.874	10.835	11.007	11.043	10.989	11.064	11.041	10.965

Bader charge of Au atoms on surface 2 {111}												
D (nm)	80	81	84	85	86	87	88	93	94	109	112	113
1.5	11.057	11.046	11.051	10.830	10.875	10.850	11.002	11.034	10.992	11.053	11.032	10.979
1.3	11.055	11.046	11.050	10.804	10.887	10.847	11.005	11.035	10.993	11.061	11.032	10.985
0.9	10.803	10.991	11.037	10.968	10.839	10.937	10.942	11.054	10.996	11.047	11.067	11.047

Supplementary Table 1. The Bader charges of two attaching Au atoms between surface 1 and surface 2.

Supplementary Notes

Note 1. Dissolution of large particles and generation of small ones

During in-situ observation, it is found that if we apply a relative high dose of electron beam ($4 - 5 \times 10^5$ e nm^{-2} s^{-1}), large gold particles are continuously dissolved while small ones generate. Then this dissolution can be terminated when the electron dose is reduced. In other words, this dissolution-precipitation process is induced by the irradiation of electron beam and can be well controlled by it. To figure out how this happens, the solution chemistry is evaluated first. The solution inside a liquid pocket is the reaction product of 2.5 ml sodium citrate aqueous solution (34 mM) and 100 ml $HAuCl_4$ aqueous solution (0.24 mM), then the result solution should include the following types of ions inside: Na^+, H^+, Cl^-, Ctr^{3-} and $AuCl_4^-$. In addition, the stoichiometry ratio of citrates and $HAuCl_4$ for production of Au nanoparticles is usually from 0.23 : 1 to 0.5 : 1[1], so most of sodium citrates are not consumed since the ratio of our original solution is 3.5 : 1. More importantly, despite the small amount of residual $AuCl_4^-$, it should possess a saturation concentration because large Au particles and ionic $AuCl_4^-$ keep a dynamic equilibrium state in this solution. On the other hand, it has been reported that the solution containing H^+ and Cl^- is a particular environment where dissolution of Au nanoparticles usually happens especially in the presence of oxidation agents[2,3]. Based on the reduction potentials, the dissolution is a spontaneous redox reaction of the electrons and ions in solution, as shown here[4,5]:

$$\mathrm{Au}^0 + 4\mathrm{H}^+ + 4\mathrm{Cl}^- + \mathrm{O}_2 + \mathrm{e}^- \rightarrow \mathrm{AuCl}_4^- + 2\mathrm{H}_2\mathrm{O} \quad E_0 = +0.227\,\mathrm{V}\ \ (\mathrm{NHE}) \qquad (1)$$

Our solution just provides such an environment to facilitate the dissolution. First of all, the TEM irradiation offers sufficient electrons for this reaction. What's more, the oxidation agents such as H_2O_2, OH and O that are produced by the interaction between incident electrons and water molecules also promote the oxidization of Au particles[6]. Therefore under electron beam irradiation, gold atoms on the surfaces of large particles are captured by Cl^- to become $AuCl_4^-$ ions and diffuse around in the solution. Consequently, these ions are reduced again by the excessive citrates to generate small particles.

Note 2. Interaction potential between gold particle pairs.

The interaction potential between two particles contains four parts:

$$U = U_{\mathrm{v}} + U_{\mathrm{m}} + U_{\mathrm{e}} + U_{\mathrm{s}} \tag{2}$$

where U_{v}, U_{s}, U_{m}, and U_{e} represent van der Waals, steric-hydration, magnetic, electrostatic and interaction potentials respectively[7,8]. For magnetic interaction U_{m}, it can be ignored because gold nanoparticles are not magnetic materials. For electrostatic interaction U_{e}, the electric dipole-dipole interaction is first evaluated. In general, binary or polynary semiconductors can exhibit large dipole moments and polarizabilities because of the non-coincidence of the centers for atoms charged negatively and positively. Such a circumstance does not hold in present case based on the fact that our particles are composed only by gold atoms. Another way to introduce dipole moments is the adsorption of opposite charges on different surfaces. It is also not applicable for our situation since citrate ions are negatively charged when adsorbed on nanoparticle surfaces[9]. And in consideration of the high symmetrical morphologies of the particles (Supplementary Fig. 5), their surface charges will not lead to effective dipole moments. However when the particle get close enough, their surface charges may undergo redistribution due to the mutual influence, and thus leading to re-arranged distribution of dipoles moments. To qualitatively analyze this dipolar re-distribution during the OA growth process, we have simulated theoretical model (two attaching Au_{79} particles with ligands at different distance), and calculated the Bader charges of the model. When the attaching Au particles get closer (Supplementary Fig. 13b), to a distance of about 1.5, 1.3 and 0.9 nm (0.9 nm is close enough in our situation. This is because when the distance reaches about 0.7 nm, the directional rotation stops and citrates detach from surfaces; and more importantly at this moment, the expulsion of surface ligands creates a transient vacuum state between the interspace of two attaching surfaces, making the pressure of surrounding liquid become the dominant force.), the Bader charges of Au atoms between surface 1 and 2 were calculated (Supplementary Fig. 13b), by which the evolution of dipole interaction between Au surfaces is qualitatively evaluated. As shown in Supplementary Table 1, when the separation distance changes from about 1.5, 1.3 and 0.9 nm, the change of Bader charge value of every atom is at most 0.1% for surface 1 and 2.3% for surface 2. It means that when the particles get closer, there is little change in the Bader charges of Au atoms on both surfaces. In other words, as the attaching crystals get close, the redistribution of dipole moments which origin from surface charges could be ignored. The results are summarized here: (1) extremely small permanent dipole moments for individual particle; (2) nearly no redistribution of dipole moments as the particles approach. We can deduce that

dipole evolution rarely plays a role in OA growth. Therefore, electric dipole-dipole interaction can be ignored in our gold particle systems, in accordance with the conclusion from other literature[10]. On the other hand, the range of electrostatic force is determined by the Debye screen length which depends on the concentrations of solution. In our system, the remnant solution inside the cell possesses high level of ionic concentrations due to the continuous evaporation of solvent during the liquid cell fabrication procedure. As discussed in Note 1, the solution contains Na^+, H^+, Cl^-, Ctr^{3-} and $AuCl_4^-$, the Debye screen length of this multi-ionic electrolyte at room temperature (293 K) can be described by the following equation[7]:

$$\kappa^{-1} = \left[\frac{\left(\rho_{\infty \mathrm{Na}} e^2 z_{\mathrm{Na}}^2 + \rho_{\infty \mathrm{H}} e^2 z_{\mathrm{H}}^2 + \rho_{\infty \mathrm{Cl}} e^2 z_{\mathrm{Cl}}^2 + \rho_{\infty \mathrm{Ctr}} e^2 z_{\mathrm{Ctr}}^2 + \rho_{\infty \mathrm{AuCl_4}} e^2 z_{\mathrm{AuCl_4}}^2 \right)}{\varepsilon_0 \varepsilon k_{\mathrm{B}} T} \right]^{-1/2} \tag{3}$$

where ρ is number density, z is the valence of ions, e is electron, ε_0 and ε are vacuum and relative permittivity respectively, k_{B} is Boltzmann constant, and T is temperature. Although the concentrations of some ions are difficult to estimate due to the evaporation of solvent in liquid cell fabrication process, the observed solid phase precipitation of NaCl crystals (Supplementary Fig. 4) suggests the solution is saturated of NaCl. Accordingly, the Debye screen length in Supplementary Equation (3) satisfies

$$\kappa^{-1} < [(\rho_{\infty \mathrm{Na}} e^2 z_{\mathrm{Na}}^2 + \rho_{\infty \mathrm{Cl}} e^2 z_{\mathrm{Cl}}^2) / \varepsilon_0 \varepsilon k_{\mathrm{B}} T]^{-1/2} = 0.304 \Big/ \sqrt{[NaCl]_{\mathrm{saturated}}} = 0.12 \text{ nm}$$

This indicates that both dipole-dipole and charge-charge interactions are screened at the separation distance larger than 0.12 nm.

As a result, the final interaction between the gold nanoparticles in our system is the steric-hydration and van der Waals potentials, described as follow[7,11]:

$$U(D) = W_0 e^{-\frac{D}{\lambda}} + \left\{ -\frac{A}{6} \left(\frac{2R^2}{(4R+D)D} + \frac{2R^2}{(2R+D)^2} + \ln \frac{(4R+D)D}{(2R+D)^2} \right) \right\} \quad (D > 0.12 \text{ nm}) \tag{4}$$

where W_0 depends on the hydration of the surfaces, D is the distance between particle surfaces, λ is the decay length, A is the Hamaker constant and R is the radius of the interacting gold particles. Apparently the combined potential between two particles is only related to their separation distance, which indicates that the work done during relative motion of the particle pair is independent with the taken path. In other words, this combined potential satisfies the definition of a typical conservative force field. Accordingly, the distribution of the separation distance should be described by Boltzmann statistics[12]:

$$P(D) = C\exp(-U(D)/k_{\mathrm{B}}T) \tag{5}$$

By adopting Supplementary Equation (4) and (5), the relationship between the separation distance and the corresponding distribution can be expressed as:

$$P(D) = C\exp\left\{-\frac{W_0}{k_{\mathrm{B}}T}e^{-\frac{D}{\lambda}} + \frac{A}{6k_{\mathrm{B}}T}\left(\frac{2R^2}{(4R+D)D} + \frac{2R^2}{(2R+D)^2} + \ln\frac{(4R+D)D}{(2R+D)^2}\right)\right\} \tag{6}$$

Supplementary Fig. 10 shows the combined separation distance distribution of 21 particle pairs during their approaching process (Figure 3a) and the corresponding fitting curve using Supplementary Equation (6). Here $R = 1.41$ nm is the mean radius of these particle pairs, C is a constant and k_{B} is Boltzmann constant. The related parameters are extracted from the fitting result: $\lambda = 0.21 \pm 0.01$ nm, $W_0 = (3.7 \pm 0.4)\times 10^{-19}$ J and $A = (4.7 \pm 0.9)\times 10^{-19}$ J. Since our steric-hydration interaction comes from two polymer covered surfaces approaching each other, it obeys $U_{\mathrm{s}} \propto e^{-\pi D/L}$ (L is the thickness of polymer layer)[7], which leads to the thickness of sodium citrate layer $L_{\mathrm{citrate}} = \pi\lambda$ (0.21 nm) = 0.66 nm. Eventually the interaction potential $U(D)$ and $F(D)$ ($F(D) = -\mathrm{d}U(D)/\mathrm{d}D$) are calculated by the obtained fitting parameters and presented in Figure 3b.

Note 3. Au nanoparticles passivated with citrate: *density functional theory (DFT)*

Computational Models and Methods

Au{111}, and Au{100} surfaces were modeled as p (4×6), p (4×4) and p (4×5) periodic slab model includes four, four and three atomic layers respectively. Atoms in the top two layers were relaxed, and all other atoms were fully frozen. The neighboring layers were separated in the direction perpendicular to the surface by a vacuum distance of 15 Å. DFT calculations were performed using projector augmented wave (PAW) potentials and the PBE functional implemented in the Vienna ab initio simulation package (VASP)[13-16]. Relaxations were carried out using conjugate-gradient algorithm, and stopped if all forces were smaller than 0.03 eV $Å^{-1}$. The kinetic-energy cutoff of plane wave was set to 400 eV. Brillouin-zone was integrated using Monkhorst-Pack-generated sets of k points. A 2×2×1 k-points grid was used for the surface calculations.

Surface energy

The surface energy (E_{surface}) was calculated as follows:

$$E_{\text{surface}} = (E^{n}_{\text{slab}} - nE_{\text{bulk}})/2A \qquad (7)$$

where E^{n}_{slab} is the total energy of the slab, E_{bulk} is the total energy of the bulk per unit cell, n is the number of bulk unit cells contained in the slab, and A is the surface area of each side for the slab. The calculated results are summarized in Supplementary Table 1.

Ligand configuration

For citrate configurations, infrared and X-ray photoelectron spectroscopy analysis have demonstrated that in aqueous solution citrates will bond with two hydrogen atoms to form dihydrogen citrate anions ($H_2Citrate^-$), which are adsorbed on gold surfaces by the central carboxylate group (η^2-COO^-)[17]. In this consideration, we propose three possible initial configurations depending on the position of oxygen atoms, described as follow:

Top configuration: the oxygen atom of central carboxylate group directly sits on the top of surface atoms.

Bridge configuration: the oxygen atom of central carboxylate group locate above the center between two surface atoms.

Hollow configuration: the oxygen atom of central carboxylate group locate above the vacancy of surface atoms.

Figure 4 and S9 show the comparison between initial and finial configurations on {111} and {100} surfaces. After relaxation, the citrate of top configuration stays still. However for

bridge and hollow configurations, the citrate spontaneously moves from its original position and develops to become the top configuration despite there is some slight deviations. This change can be observed on both these two surfaces, suggesting that the top configuration is energetically most stable state for adsorbed citrates.

Binding energy

The adsorption/binding energy (E_{Binding}) of citrate radical ($H_2Citrate^-$) on the Au {111} and Au {100} was investigated, which was defined as:

$$E_{\text{Binding}} = E_{\text{Surface}} + E_{\text{H}_2\text{Citrate}^-} - E_{\text{H}_2\text{Citrate}^-/\text{Surface}} \tag{8}$$

where E_{Surface}, $E_{\text{H}_2\text{Citrate}^-}$ and $E_{\text{H}_2\text{Citrate}^-/\text{Surface}}$ are the energies of the surface, citrate radical and citrate radical adsorbed on the surface respectively. Each individual energy term on the right side can be achieved directly from DFT calculation.

Bader charges

In this part, the model has been simplified to the configuration that two citrate ligands symmetrically adsorb on {111} surfaces of Au_{79} particles (with the size of 1.3 nm), as shown in Supplementary Fig. 13a. Then the Bader charges are calculated when they get closer.

Note 4. Comparison of OA trajectories between our results and other studies.

Trajectories of OA in solution have been in-situ investigated for Pt_3Fe[18] and Fe_2O_3[19]. The difference between our phenomenon and previous publications should be emphasized here.

In our previous work[18], it is found that polycrystalline nano-chains can be built by imperfect attachment of nanoparticles when the dipolar interaction compels the particles to arrange end-by-end. In that work, the drift velocity increases greatly at very close distance because the attractive dipolar interaction ($U \sim 1/r^2$) increases dramatically as the particles get closer. This increase of dipolar interaction also leads to an apparent acceleration process when the distance decreases to 3 nm for particle-particle pair and 6 nm for particle-chain pair (Figure 4A, *Science* 2012, 336, 1011). However, the situation is different in this work. The attractive force does not change much as the particles get closer, and then even becomes repulsive force when the distance is smaller than about 1 nm (see the dashed magenta curves in Figure 3b, this manuscript). This is why the drift velocity slightly decreases as they closer (Supplementary Fig. 14). In addition, the abrupt increase of velocity before the final contact (Supplementary Fig. 14) well indicates that the expulsion of surface ligands leads to the vanishing of repulsive force and then quick contact. Therefore the jump to contact in this work should be distinguished from the increase of drift velocity in our previous work, similar behavior but different mechanism.

In another previous work[19], the pathways of OA in solution has been in-situ observed. Although the authors have imaged the successive pre-alignment, jump to contact and interface elimination process, they have not performed quantitatively statistical analysis about the detailed evolvement laws of particle's movement and rotation before contact. In comparison, here in our work, the small size of gold particles allows us to study the movement and rotation behavior more easily, and a large number of statistical data also allow us to investigate the nature of driving force. More details about OA have been discovered in our work.

Most importantly, numerous literature about nano-synthesis have noticed the crucial role of surface ligands in OA, whereas there is still no direct evidence to elucidate this mechanism. Our results have, for the first time, revealed how the surface ligands control OA and provided a new mechanism of the binding energy related crystal facet selection. These new findings were enabled by the liquid cell TEM with significant improved spatial resolution as compared to the previous reports.

Supplementary References

1 Leng, W., Pati, P. & Vikesland, P. J. Room temperature seed mediated growth of gold nanoparticles: mechanistic investigations and life cycle assesment. *Environ. Sci.: Nano* **2**, 440-453 (2015).

2 Shi, H., Bi, H., Yao, B. & Zhang, L. Dissolution of Au nanoparticles in hydrochloric acid solution as studied by optical absorption. *Appl. Surf. Sci.* **161**, 276-278 (2000).

3 Tsuji, M. *et al.* Shape and size controlled synthesis of gold nanocrystals using oxidative etching by $AuCl_4^-$ and Cl^- anions in microwave-polyol process. *Coll. Surf. A* **302**, 587-598 (2007).

4 Rodríguez-Fernández, J., Perez-Juste, J., Mulvaney, P. & Liz-Marzan, L. M. Spatially-directed oxidation of gold nanoparticles by Au(III)-CTAB complexes. *J. Phys. Chem. B* **109**, 14257-14261 (2005).

5 Soejima, T. & Kimizuka, N. One-pot room-temperature synthesis of single-crystalline gold nanocorolla in water. *J. Am. Chem. Soc.* **131**, 14407-14412 (2009).

6 Garrett, B. C. *et al.* Role of water in electron-initiated processes and radical chemistry: issues and scientific advances. *Chem. Rev.* **105**, 355-390 (2005).

7 Israelachvili, J. N. Intermolecular and surface forces: revised third edition. (Academic press, 2011).

8 Bishop, K. J., Wilmer, C. E., Soh, S. & Grzybowski, B. A. Nanoscale forces and their uses in self-assembly. *Small* **5**, 1600-1630 (2009).

9 López-Lorente, Á. I., Soriano, M. L. & Valcárcel, M. Analysis of citrate-capped gold and silver nanoparticles by thiol ligand exchange capillary electrophoresis. *Microchim. Acta* **181**, 1789-1796 (2014).

10 Tang, Z., Zhang, Z., Wang, Y., Glotzer, S. C. & Kotov, N. A. Self-assembly of CdTe nanocrystals into free-floating sheets. *Science* **314**, 274-278 (2006).

11 Mondain-Monval, O. *et al.* Polymer-induced repulsive forces: exponential scaling. *Phys. Rev. Lett.* **80**, 1778 (1998).

12 Anand, U., Lu, J., Loh, D., Aabdin, Z. & Mirsaidov, U. Hydration layer-mediated pairwise interaction of nanoparticles. *Nano Lett.* **16**, 786-790 (2016).

13 Kresse, G. & Hafner, J. Ab initio molecular-dynamics simulation of the liquid-metal–amorphous-semiconductor transition in germanium. *Phys. Rev. B* **49**, 14251 (1994).

14 Kresse, G. & Furthmüller, J. Efficiency of ab-initio total energy calculations for metals and semiconductors using a plane-wave basis set. *Comp. Mater. Sci.* **6**, 15-50 (1996).

15 Blöchl, P. E. Projector augmented-wave method. *Phys. Rev. B* **50**, 17953 (1994).

16 Kresse, G. & Furthmüller, J. Efficient iterative schemes for ab initio total-energy calculations using a plane-wave basis set. *Phys. Rev. B* **54**, 11169 (1996).

17 Park, J.-W. & Shumaker-Parry, J. S. Structural study of citrate layers on gold nanoparticles: role of intermolecular interactions in stabilizing nanoparticles. *J. Am. Chem. Soc.* **136**, 1907-1921 (2014).

18 Liao, H. G., Cui, L., Whitelam, S. & Zheng, H. Real-time imaging of Pt_3Fe nanorod growth in solution. *Science* **336**, 1011-1014 (2012).

19 Li, D. *et al.* Direction-specific interactions control crystal growth by oriented attachment. *Science* **336**, 1014-1018 (2012).

20 Li, Z. *et al.* Three-dimensional atomic-scale structure of size-selected gold nanoclusters. *Nature* **451**, 46-48 (2008).

21 Cleveland, C. L. *et al.* Structural evolution of smaller gold nanocrystals: the truncated decahedral motif. *Phys. Rev. Lett.* **79**, 1873 (1997).

22 Li, H. *et al.* Magic-number gold nanoclusters with diameters from 1 to 3.5 nm: Relative stability and

catalytic activity for CO oxidation. *Nano Lett.* **15**, 682-688 (2014).
