# Supplementary material for: In-situ liquid cell transmission electron microscopy investigation on oriented attachment of gold nanoparticles
Source: Nat Commun. 2018 Jan 29;9:421. doi: 10.1038/s41467-018-02925-6 (PMC5788991; doi:10.1038/s41467-018-02925-6)
Supplement: Supplementary file 3 — Description of Additional Supplementary Files [file 41467_2018_2925_MOESM3_ESM.pdf]

Description of Supplementary Files

File Name: Supplementary Movie 1
Description: The dissolution of large gold particles under electron beam irradiation and generation of small particles in the meantime.

File Name: Supplementary Movie 2
Description: The motion and dissolution behaviours of large gold particles inside a liquid pocket.

File Name: Supplementary Movie 3
Description: Oriented attachment trajectories of two small gold particles, which finally form a single particle with twin interface at their contact surfaces.

File Name: Supplementary Movie 4
Description: Oriented attachment trajectories of two small gold particles, which finally form a monocrystalline particle.
